# Analysis of the genetic variants associated with circulating levels of sgp130. Results from the IMPROVE study

**DOI:** 10.1038/s41435-019-0090-z

**Published:** 2020-01-14

**Authors:** Alice Bonomi, Fabrizio Veglia, Damiano Baldassarre, Rona J. Strawbridge, Zahra Golabkesh, Bengt Sennblad, Karin Leander, Andries J. Smit, Philippe Giral, Steve E. Humphries, Elena Tremoli, Anders Hamsten, Ulf de Faire, Bruna Gigante, on behalf of the IMPROVE study group, C. R. Sirtori, S. Castelnuovo, L. Calabresi, M. Amato, B. Frigerio, A. Ravani, D. Sansaro, D. Coggi, C. C. Tedesco, P. Eriksson, A. Silveira, F. Laguzzi, J. Cooper, J. Acharya, K. Huttunen, E. Rauramaa, H. Pekkarinen, I. M. Penttila, J. Törrönen, R. Rauramaa, A. I. van Gessel, A. M. van Roon, G. C. Teune, W. D. Kuipers, M. Bruin, A. Nicolai, P. Haarsma-Jorritsma, D. J. Mulder, H. J. G. Bilo, G. H. Smeets, J. L. Beaudeux, J. F. Kahn, V. Carreau, A. Kontush, J. Karppi, T. Nurmi, K. Nyyssönen, R. Salonen, T. P. Tuomainen, J. Tuomainen, J. Kauhanen, S. Kurl, G. Vaudo, A. Alaeddin, D. Siepi, G. Lupattelli, E. Mannarino

**Affiliations:** 1 Centro Cardiologico Monzino, IRCCS, Milan, Italy; 2 Department of Medical Biotechnology and Translational Medicine, Università degli Studi di Milano, Milan, Italy; 3 Institute of Health and Wellbeing, University of Glasgow, Glasgow, UK; 4 Department of Medicine Solna, Cardiovascular Medicine Unit, Karolinska Institutet, Stockholm, Sweden; 5 Unit of Translational Epidemiology, Institute of Environmental Medicine, Karolinska Institutet, Stockholm, Sweden; 6 National Bioinformatics Infrastructure Sweden, Science for Life Laboratory, Uppsala University, Uppsala, Sweden; 7 Unit of Cardiovascular and Nutritional Epidemiology, IMM, Karolinska Institutet, Stockholm, Sweden; 8 Department of Medicine, University Medical Center Groningen and University of Groningen, Groningen, The Netherlands; 9 Unités de Prévention Cardiovasculaire, Assistance Publique-Hôpitaux de Paris, Service Endocrinologie-Metabolisme, Groupe Hôpitalier Pitie-Salpetriere, Paris, France; 10 Centre for Cardiovascular Genetics, University College London, London, UK; 11 Dipartimento di Scienze Farmacologiche e Biomolecolari, Università di Milano, Milan, Italy; 12 Department of Medicine, Cardiovascular Medicine Unit, Karolinska Institutet and Karolinska University Hospital Solna, Stockholm, Sweden; 13 Unit of Cardiovascular and Nutritional Epidemiology, Institute of Environmental Medicine, Karolinska Institutet, Stockholm, Sweden; 14 Department of Medicine, Rayne Institute, University College of London, London, UK; 15 Foundation for Research in Health Exercise and Nutrition, Kuopio Research Institute of Exercise Medicine, Kuopio, Finland; 16 Department of Medicine, University Medical Center Groningen, Groningen, The Netherlands; 17 Department of Medicine, Isala Clinics Zwolle, Zwolle, The Netherlands; 18 Institute of Public Health and Clinical Nutrition, University of Eastern Finland, Kuopio Campus, Kuopio, Finland; 19 Internal Medicine, Angiology and Arteriosclerosis Diseases, Department of Clinical and Experimental Medicine, University of Perugia, Perugia, Italy

**Keywords:** Cytokines, Genetic variation

## Abstract

The genes regulating circulating levels of soluble gp130 (sgp130), the antagonist of the inflammatory response in atherosclerosis driven by interleukin 6, are largely unknown. Aims of the present study were to identify genetic loci associated with circulating sgp130 and to explore the potential association between variants associated with sgp130 and markers of subclinical atherosclerosis. The study is based on IMPROVE (*n* = 3703), a cardiovascular multicentre study designed to investigate the determinants of carotid intima media thickness, a measure of subclinical atherosclerosis. Genomic DNA was genotyped by the CardioMetaboChip and ImmunoChip. About 360,842 SNPs were tested for association with log-transformed sgp130, using linear regression adjusted for age, gender, and population stratification using PLINK v1.07. A *p* value of 1 × 10^−5^ was chosen as threshold for significance value. In an exploratory analysis, SNPs associated with sgp130 were tested for association with c-IMT measures. We identified two SNPs significantly associated with sgp130 levels and 24 showing suggestive association with sgp130 levels. One SNP (rs17688225) on chromosome 14 was positively associated with sgp130 serum levels (*β* = 0.03 SE = 0.007, *p* = 4.77 × 10^−5^) and inversely associated with c-IMT (c-IMT_mean–max_ *β* = −0.001 SE = 0.005, *p* = 0.0342). Our data indicate that multiple loci regulate sgp130 levels and suggest a possible common pathway between sgp130 and c-IMT measures.

## Introduction

The soluble gp130 (sgp130) is a master regulator of cytokine-mediated inflammatory, regenerative, and proliferative effects [1–3]. Three main sgp130 isoforms, with molecular weights between 50 and 110 KDa, can be detected in the circulation: sgp130-RAPS [4], sgp130-E10 [5], and full length sgp130 [6] produced by alternative splicing, alternative intronic polyadenylation [5], and shedding of the membrane gp130 receptor in a cell specific manner [3]. Biological assays commonly used to measure sgp130 do not differentiate among these three isoforms.

The main role of circulating sgp130 is anti-inflammatory. Sgp130 has a high affinity (1 mM) for IL6:sIL6R, the complex that drives the pro-inflammatory and the pro-atherogenic IL6 trans-signaling pathway [7, 8]. Binding of sgp130 to IL6:sIL6R results in neutralization of the complex [9] thus blunting the inflammatory response. It was recently shown in in vitro condition that the full length sgp130 is the most potent inhibitor of IL6 trans-signaling [3]. A recombinant form of sgp130 (sgp130Fc) has been shown to be exert an atheroprotective effect in a mouse experimental model of atherosclerosclerosis [10] and potentially able to antagonize the pro-inflammatory effect driven by IL11 trans-signaling [11].

Clinical [12] and experimental evidence [10, 13] suggest causality of IL6 trans-signaling on the inflammatory response in atherosclerosis and data from our group indicate that an excess of the circulating IL6:sIL6R over the ternary IL6:sIL6R:sgp130 complex increases the risk for future cardiovascular (CV) events [14].

The genes regulating sgp130 levels are largely unknown. One single-nucleotide polymorphism (rs2228044) in *GP130* (chromosome 5) encoding an amino acid change Gly148Arg, has been shown to be associated with lower sgp130 circulating levels [15] and a reduced risk of myocardial infarction [16]. Given the central role of sgp130 in orchestrating the inflammatory response in atherosclerosis, knowledge of the genes regulating sgp130 circulating levels might provide novel insights on the mechanisms underlying its synthesis and release and also suggest if sgp130 might represent a novel therapeutic moiety to modulate the inflammatory response in atherosclerosis.

The aim of the present study was to identify SNPs associated with serum levels of sgp130, using genetic data from the carotid Intima Media Thickness (c-IMT) and c-IMT Progression as Predictors of Vascular Events (IMPROVE), a high cardiovascular risk European population study. In secondary analysis, genetic variants associated with sgp130 were tested for association with c-IMT, a measure of vascular wall remodeling indicative of subclinical atherosclerosis.

## Results

Table 1 summarizes the characteristics of the IMPROVE study participants included in the present study according to sgp130 quartiles. High sgp130 levels were more often observed in women and in study participants with diabetes and hypercholesterolemia.

**Table 1:** Baseline characteristics of the IMPROVE study participants included in the study according to the sgp130 quartiles.

	Sgp130 Q1 (*n* = 859)	Sgp130 Q2 (*n* = 860)	Sgp130 Q3 (*n* = 860)	Sgp130 Q4 (*n* = 860)
Age (years)	64.52 ± 5.19	64.69 ± 5.47	63.88 ± 5.41	63.63 ± 5.57
Male *N* (%)	485 (27.67)	432 (24.64)	441 (25.16)	395 (22.53)
BMI (kg/cm^2^)	27.11 ± 4.25	27.17 ± 4.1	27.33 ± 4.25	27.46 ± 4.46
Waist/hip (cm)	0.92 ± 0.09	0.92 ± 0.08	0.92 ± 0.09	0.91 ± 0.08
SBP (mmHg)	141.47 ± 19.37	142.77 ± 18.35	142.08 ± 18.77	141.58 ± 17.36
DBP (mmHg)	81.74 ± 10.07	82.13 ± 9.58	81.76 ± 9.81	82.27 ± 9.65
Risk factors for cardiovascular disease *N* (%)				
Smoking	148 (17.23)	116 (13.49)	125 (14.53)	127 (14.77)
Hypercholesterolemia	658 (76.60)	661 (76.86)	668 (77.67)	692 (80.47)
Hypertension	712 (77.73)	743 (81.11)	733 (79.93)	723 (78.93)
Diabetes	228 (25.39)	226 (25.06)	256 (28.60)	255 (28.02)
Biochemical measurements				
LDL-cholesterol (mmol/L)	3.54 ± 0.98	3.55 ± 1.03	3.53 ± 1.01	3.57 ± 1
Glucose (mmol/L)	5.97 ± 1.51	5.91 ± 1.58	5.93 ± 1.68	5.85 ± 1.75
Creatinine (micromol/L)	80.23 ± 17.26	81.27 ± 17.88	81.19 ± 17.76	80.94 ± 18.1
Inflammatory biomarkers				
C reactive Protein (mg/L)	1.74 (0.73–3.45)	1.73 (0.71–3.47)	1.88 (0.74–3.61)	2.08 (0.89–3.93)
Sgp130 (ng/ml)	382.75 ± 53.84	507.30 ± 33.76	632.06 ± 40.73	837.08 ± 113.13

Missing values: BMI, *n* = 1; waist/hip ratio, *n* = 10; SBP and DBP, *n* = 4; diabetes, *n* = 54; LDL-cholesterol, *n* = 68; glucose, *n* = 7; creatinine, *n* = 7; C-reactive protein, *n* = 2

*BMI* body mass index, *SBP* systolic blood pressure, *DBP* diastolic blood pressure, *LDL* low-density lipoprotein

### Genetic variants associated with serum sgp130 levels

Table 2 summarizes the SNPs with significant or suggestive associations with serum sgp130 levels after adjustment for age, sex, and population structure. Supplementary Fig. II displays the Manhattan plot summarizing the results of the association analysis.

**Table 2:** SNPs associated with circulating serum sgp130 levels.

Chr	SNP	EA	Frequency (%)	*β*		SE	*P*	Gene	Contig/gene sequence	Functional consequence
Significant (*p* value < 1 × 10^−5^)										
3	rs10935473	T	47	−0.014		0.003	9.45 × 10^−6^	Unknown	NT_005612.17	–
10	rs1929666	T	10	0.025		0.005	1.63 × 10^−6^	*LOC105378515*	NT_030059.14	Intronic SNP
Suggestive (*p* value < 1 × 10^−4^)										
1	rs74760246	T	7	−0.028		0.006	1.21 × 10^−5^	*CRB1*	NG_008483.2	Intronic SNP
1	rs3006246	A	26	−0.015		0.003	4.31 × 10^−5^	*NR5A2*	NM_001276464.1	Intronic SNP
3	rs9858592	C	49	−0.013		0.003	5.62 × 10^−5^	*ST3GAL6AS1*	NR_046683.1	Intronic SNP
5	rs2228043	C	13	0.019		0.004	9.81 × 10^−5^	*GP130*	NM_001190981.1	NS aa change L397V
7	rs2622168	A	3	0.041		0.010	4.37 × 10^−5^	*DPP6*	NT_007933.16	Intronic SNP
7	rs73063812	C	5	−0.030		0.007	7.27 × 10^−5^	*DGKB*	NM_004080.2	3′UTR
7	rs11767669	A	15	−0.018		0.004	3.92 × 10^−5^	Unknown	NT_007933	–
8	rs3087409	A	5	0.029		0.007	2.70 × 10^−5^	*WRN*	NG_008870.1	Intronic SNP
9	rs12379461	A	36	−0.013		0.003	9.25 × 10^−5^	*OBP2B*	NT_008470.20	–
9	rs16932962	C	6	0.027		0.007	9.09 × 10^−5^	*TTC39B*	NM_001168339.1	Intronic SNP
10	rs1972396	T	3	0.035		0.008	7.72 × 10^−5^	*CACNB2*	NM_000724.3	Intronic SNP
11	rs1681503	T	2	0.043		0.010	4.62 × 10^−5^	*ARAP1*	NM_001040118.2	Intronic SNP
12	rs6582091	A	3	−0.039		0.010	8.87 × 10^−5^	*TRHDE*	NM_013381.2	Intronic SNP
13	rs11069292	G	15	−0.019		0.004	4.06 × 10^−5^	*LOC105370328*	XR_931670	Intronic SNP
13	rs9529615	A	37	0.013		0.003	6.40 × 10^−5^	Unknown	NT_024524	–
14	rs17688225	A	5	0.030		0.007	4.77 × 10^−5^	Unknown	NC_000014.7	
14	rs12886000	T	15	0.017		0.004	6.93 × 10^−5^	*LOC107984706*	XR_001750873.1	–
17	rs1872083	T	30	−0.014		0.003	4.63 × 10^−5^	*SDK2*	NM_001144952.1.	Intronic SNP
17	rs4795780	T	21	0.015		0.003	6.10 × 10^−5^	*ASIC 2*	NM_001144952.1.	Intronic SNP
17	rs2955617	A	32	0.014	0.003		2.43 × 10^−5^	Unknown	NT_010718.17	–
19	rs3813774	A	6	−0.028		0.006	4.63 × 10^−5^	*FBN3*	NM_001321431.1	S aa change C643C
20	rs4809631	C	17	−0.019		0.004	1.75 × 10^−5^	*ZMYND8*	NM_001281771	Intronic SNP
20	rs35425776	A	97/3	0.038	0.008		1.09 × 10^−5^	Unknown	NT_011362.11	–
20	rs808682	T	75/25	−0.015	0.003		8.78 × 10^−5^	Unknown	NT_011387	–

*Chr* chromosome, *EA* effect allele, *S* synonymous, *NS* non synonymous, *Aa* amino acid, *CRB1* crumbs 1, cell polarity complex component*, NR5A2* nuclear receptor subfamily 5 group A member 2, *ST3GAL6AS1* ST3GAL6 antisense RNA 1, *GP130* glycoprotein 130, *L* Leucin, *V* Valin, *DPP6* dipeptidyl peptidase like 6, *DGKB* diacylglycerol kinase beta, *WRN* Werner syndrome RecQ like helicase, *OBP2B* odorant binding protein 2B, *TTC39B* tetratricopeptide repeat domain 39B, *3*′*UTR* 3′ untraslated region, *LOC* uncharacterized locus, *CACNB2* calcium voltage-gated channel auxiliary subunit beta 2, *ARAP1* ArfGAP with RhoGAP domain, ankyrin repeat and PH domain 1, *TRHDE* thyrotropin releasing hormone degrading enzyme, *SDK2* sidekick cell adhesion molecule 2, *ASIC2* acid sensing ion channel subunit 2, *SLC14A2* solute carrier family 14 member 2, *FBN3* fibrillin 3, *C* cysteine, *ZMYND8* zinc finger MYND-type containing 8

**Fig. 1:**
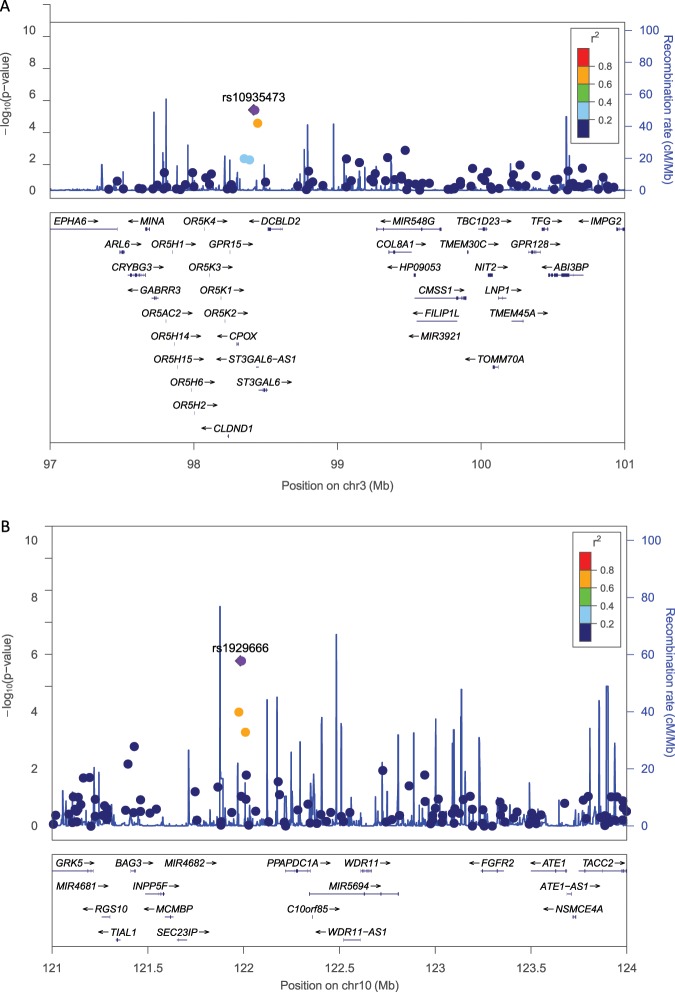
Regional association plot of chromosome 3 and chromosome 10 (Chr10) loci. Regional association plot of chromosome 3 (Chr3) (Panel A) and chromosome 10 (Chr10) (Panel B) loci. The diamond (shown in purple) corresponds to the index SNP identified as associated with sgp130, rs10935473 on chromosome 3, and rs1929666 on chromosome 10. The SNPs in the region are represented by circles. The color of the circle exemplifies the degree of LD with the index SNP (see *R*^2^ values on the right of the figure).

According to the significance threshold value we chose, only two SNPs were significantly associated with circulating sgp130 levels: rs10935473 (on chromosome 3, Fig. 1a) and rs1929666 (on chromosome 10, Fig. 1b).

Rs10935473 is in moderate linkage disequilibrium (LD) (*r*^2^ : 0.67) with rs9858592 located in the *ST3GAL6-anti sense RNA 1* (*ST3GAL6AS1*) (Table 2). The GTEx expression panel reports the effect allele (EA) at both SNPs as associated with a lower expression of the long noncoding RNA *ST3GAL6* in a large panel of tissues such as the adipose tissue, the heart, and the arterial wall (https://gtexportal.org/home/snp/rs10935473) and with lower levels of circulating sgp130.

Among the SNPs potentially associated with sgp130 serum levels, we have identified a potentially functional SNP, rs2228043, which encodes a missense amino acid change L370V in *GP130* (chromosome 5). This SNP maps to the coding region of *GP130* isoform 1(NM_002184.4) (exon 10) and to the 3′UTR of *GP130* isoform 2 (NM_175767.3), known also as gp130-RAPS. The GTEx expression panel reports a lower tissue gp130 expression in the tibial nerve in the heterozygote GC, while too few observations are available for the GG genotype group to define the direction of the effect (https://www.gtexportal.org/home/snp/rs2228043).

Only two of the SNPs identified in the present study have formerly been associated with the risk of inflammatory and CV diseases: rs74760246 (chromosome 1), in the intronic region of *CRB1*, is in strong LD (*r*^2^ ≥ 0.8) with rs1421389 and rs10494757 mapping at *DENNB1*, a gene associated with the risk of chronic inflammatory diseases [17, 18]; rs3087409 (chromosome 8) at *WRN*, an intronic SNP in full LD with a variant previously associated with premature aging and with the risk of myocardial infarction and stroke [19].

The other SNPs identified as suggestively associated with sgp130 circulating levels can be grouped in SNPs mapping at genetic loci previously associated with the regulation of cholesterol and glucose metabolism such as rs3006246 (chromosome 1) in *NR5A2*, also known as liver receptor homolog 1 [20], rs3813774 in *FBN3* (chromosome 19) an SNP causing a synonymous amino acid change and rs73063812 (chromosome 7) in *DKGB* 3′UTR all inversely associated with circulating sgp130 levels and rs1681503 (chromosome 11) in *ARAP1* [21] and rs16932962 (chromosome 9) in *TTC39B* positively associated with sgp130*. TTC39B* has unknown function, however SNPs mapping at this gene, in low LD (*r*^2^) with the SNPs identified here have been associated with low HDL levels [22]. Finally, rs6582091 (chromosome 12) in *TRHDE* a metallopeptidase 1 involved in the degradation of thyrotropin differentially expressed in the perivascular and subcutaneous fat [23].

In addition, some suggestive SNPs map to loci encoding auxiliary subunits of membrane ion channels, such as rs2622168 (chromosome 7) in *DPP6* (a dipeptidyl peptidase that enhances expression and kinetics of voltage-gated K(+) channels on muscular cells and neurons [24]) and rs1972396 (chromosome 10) in *CACNB2* (encoding a subunit of calcium voltage-gated [25]) and rs4795780 at *ASIC 2* (chromosome 17) (encoding an amiloride-sensitive sodium channel).

Taken together the 26 SNPs explained 11% of the variance in circulating sgp130 levels, while each single SNP explained less than 1% of the total variance.

### Secondary analysis: association of the SNPs associated with sgp130 with c-IMT measures

We performed an exploratory analysis where the SNPs with significant or suggestive associations with sgp130 were tested for association with measures of c-IMT at baseline.

Three SNPs were nominally associated (*p* value < 0.05) with measures of c-IMT as shown in Table 3. After adjustment for age, sex, multidimensional scaling (MDS), and sgp130, only rs17688225 on chromosome 14 remained negatively associated with c-IMT measures at baseline (c-IMT_mean_: *β* = −0.010 SE = 0.005, *p* = 0.0251; c-IMT_mean–max_: *β* = −0.010 SE = 0.005, *p* = 0.0347; c-IMT_max_: *β* = −0.025 SE = 0.009, *p* value = 0.0049). Of interest, this allele is positively associated with levels of sgp130 (*β* = 0.030 SE = 0.007, *p* value = 4.77 × 10^−5^).

**Table 3:** SNPs associated with c-IMT measures at baseline.

	Model 1			Model 2		
	*β*	SE	*p* value	*β*	SE	*p* value
c-IMT_mean_						
rs17688225	−0.010	0.005	0.0327	−0.010	0.005	0.0251
rs4809631	−0.003	0.003	0.2179	−0.003	0.003	0.2636
c-IMT_max_						
rs17688225	−0.024	0.009	0.0074	−0.025	0.009	0.0049
rs4809631	−0.010	0.005	0.0381	−0.010	0.005	0.0537
c-IMT_mean–max_						
rs17688225	−0.010	0.005	0.0422	−0.010	0.005	0.0342
rs4809631	−0.004	0.003	0.1525	−0.004	0.003	0.1819
rs3813774	−0.007	0.005	0.1473	−0.006	0.005	0.1772

*β* beta, *SE* standard error

Model 1: Adjusted for age, sex and latitude

Model 2: Model 1 + sgp130

## Discussion

This is the first study presenting a systematic analysis of the genetic variants associated with circulating sgp130 in a large European population. We have identified multiple SNPs, each one exerting a small effect on circulating levels of sgp130. Most of the SNPs identified showed a weak association with circulating levels of sgp130 and only two SNPs (rs10935473 and rs1929666) surpassed the prespecified significance threshold level. The large number of variants regulating sgp130 probably reflect its pleiotropic effect in a large spectrum of chronic inflammatory and autoimmune diseases [26] and has been also observed in other studies analyzing the genetic basis of complex phenotypes [27].

Our results indicate that a genetic locus on chromosome 3 might be relevant for the regulation of circulating levels of sgp130. One of the SNPs identified in our study (rs9858592) is in strong LD (*r*^2^ > 0.8) with two intronic *ST3GAL6AS1* SNPs (rs4857414 and rs12635955) previously reported on the NCBI database to be associated with circulating sgp130 (https://www.ncbi.nlm.nih.gov/projects/SNP/GaPBrowser_prod/callGaPBrowser2.cgi?snp=828588&aid=3748). *ST3GAL6AS1* codes for a long noncoding RNA, possibly involved in the regulation of the expression of a sialyltransferase, *ST3GAL6* [28]. Sialylation contributes to regulation of cell adhesion and is recognized as one of the cellular mechanisms promoting atherosclerosis [29]. The role of the antisense RNA identified as a regulator of sgp130 has not been defined in atherosclerosis. Rs9858592 is in moderate LD (*r*^2^ = 0.69) with rs865474, another SNP in *ST3GAL6* previously reported as causally associated with body mass index [30].

Individuals with metabolic syndrome demonstrated elevated sgp130 levels [31] and additional nine SNPs located at genetic loci involved in the regulation of glucose and lipid metabolism, as well as associated with obesity, have been identified as potentially associated with circulating sgp130 levels in the present study. Taken together our data suggest that variants regulating sgp130 levels are also involved in the regulation of cardiometabolic phenotypes where a low-grade inflammation is commonly observed.

Among the SNPs showing a suggestive association with sgp130 we report rs2228043, in *GP130*. Rs2228043 is in full LD (*r*^2^ = 0.99) with rs2228044. The EA at both SNPs associates with higher sgp130 levels [15]. Rs2228043 introduces a Leu397Val amino acid substitution in exon 10 while rs2228044 introduces a Gly148Arg amino acid substitution in exon 5, both in the extracellular part of the protein which is formed by six fibronectin-type III-like domains [32] (https://www.uniprot.org/uniprot/P40189). Exon 5 belongs to the second fibronectin-type III-like domain, a region contributing to regulate the efficiency of the binding to circulating cytokine [33, 34]; while exon 10, is proximal to the gp130 transmembrane region and necessary for an effective gp130 signal transduction [35]. The mechanisms underlying the association of these genetic variants with circulating sgp130 are unknown and deserve further investigations. However, one might speculate that these mutations may change the conformation and/or stability of the extracellular domain and by doing so they may favor the shedding of the membrane-bound gp130.

Another group of SNPs possibly associated with sgp130 map at loci encoding regulatory subunits of voltage-gated channels previously associated with the risk of cardiac arrhythmias [36–38], neurodegenerative [39] and psychiatric disorders [40, 41], and telomere length [42]. Functional studies have indicated that a cross-talk between the IL6 signaling and voltage-gated channels participates in the regulation of nociception in response to trauma or inflammatory disease [43] such as rheumatoid arthritis [44].

In our secondary analyses we have identified one SNP associated negatively with c-IMT measures at baseline and positively with levels of sgp130. The candidate gene at this locus is unclear. The opposite direction of these associations is consistent with a protective effect of sgp130 in atherosclerosis, which has previously been demonstrated: high levels of sgp130 exert a protective effect on the atherosclerotic process as shown by data obtained in a mouse experimental model of atherosclerosis where treatment with recombinant sgp130 was associated with regression of atherosclerotic lesions [10].

This study has several limitations. It is an observational study and as such we cannot provide insights on the mechanisms underlying the observed associations, nor can the causality of sgp130 on atherosclerosis be assessed. The IMPROVE is a multicentre study where study participants had high risk for CV events, which hampers the generalization of our results to the general population. The important strengths of the current study are the use of standardized methods across the recruitment sites and genetic data with prior probability of associations with cardiometabolic, immune, or inflammatory conditions.

In conclusion, we report here the first systematic investigation of the genetic variants associated with circulating levels of sgp130, the natural antagonist of the IL6 trans-signaling. Our results indicate that multiple genetic loci participate in the regulation of sgp130 levels, some possibly overlap with those regulating c-IMT measures and highlight a number of cardiometabolic pathways in which sgp130 might participate. This study suggests that investigation of the causality of sgp130 in atherosclerosis would be of value, as this is a prerequisite for identifying novel molecular drug targets.

## Materials/subjects and methods

### Study population

The IMPROVE study is a European multicentre, longitudinal, observational study, fully described elsewhere [45]. Briefly, from March 2004 to April 2005 seven different centers in five European Countries (Italy, France, The Netherlands, Sweden, and Finland) recruited 3711 study participants (age 54–79 years) with at least three vascular risk factors [i.e., men, women at least 5 years after menopause, dyslipidemia, hypertension, diabetes, smoking, and family history of CV disease] but without diagnosed CV and/or cerebrovascular disease. At enrollment, study participants filled in an extensive questionnaire on medical history, life style habits, CV risk factors, co-morbidities, current, and past medications and underwent a medical assessment where anthropometric measures and blood pressure were measured and recorded. Smoking was defined as current smoking. Hypertension was defined as self-reported and/or diastolic blood pressure (DBP) ≥ 90 mmHg and/or systolic blood pressure (SBP) ≥ 140 mmHg and/or treatment with antihypertensive drugs; diabetes was defined as self-reported and/or blood glucose level ≥ 7 mmol/L and/or treatment with insulin or oral hypoglycaemic drugs. Hypercholesterolemia was defined as LDL cholesterol ≥ 4.13 mmol/L and/or treatment with cholesterol lowering drugs.

Blood samples were collected after an overnight fast and stored at −80 °C until analysis.

A detail description of the protocol, the validation and the precision of carotid ultrasound measurements has been reported elsewhere [45–47]. Ultrasonographic measures of the carotid arteries were recorded at baseline by measuring four consecutive segments at the far wall of from each carotid artery. Data from the eight segments in each patient were averaged to estimate the c-IMT_mean_, c-IMT_max_, and c-IMT_mean–max_. Data are expressed in mm.

### Selection of SNPs, genotyping, and quality control procedure

Genomic DNA from IMPROVE study participants was genotyped with two genotyping arrays, the CardioMetaboChip 200k and the ImmunoChip, each one containing ~200,000 genetic variants [48, 49]. The CardioMetaboChip 200 K is a custom Illumina iSelect genotyping array including genetic variants mapping in genetic regions identified in genome-wide association (GWA) studies as potentially relevant for cardiometabolic diseases [49]. The Immonochip is a custom Illumina Infinium HD array designed to densely genotype immune-mediated diseases using loci identified by GWA studies [48].

Standard quality control procedures for genetic data were conducted on the individual genotyping chip as well as the combined dataset. MDS components were calculated using PLINK v1.07 [50] to identify possible non-European ethnicity and to enable adjustment for population structure. Three MSD components were found to be informative (MSD1, MSD2, and MSD3). One-hundred and eleven study participants did not have genotype data. SNPs were excluded if deviation from Hardy–Weinberg equilibrium (*p* < 0.0000001), call rate <95% or minor allele frequency (MAF) <1% was detected. Subjects were excluded due to cryptic relatedness, ambiguous sex or if they were identified as outliers by MDS analysis (*n* = 86). After exclusions, a total of 360,842 SNPs and 3439 study participants were available for genetic analysis. Supplementary Fig. I summarizes the exclusion criteria applied in the present study and the total number of study participants included in the analysis.

### Sgp130 measurement

Serum samples were missing for 67 subjects. Serum levels of sgp130 were measured by the Human sgp130 DuoSet ELISA development kit (#DY228) provided by R&D Systems® (R&D systems Minneapolis, MN, USA) using a protocol previously reported [51].

### Statistical analysis

Continuous variables with a normal distribution are presented as mean ± SD while variables with a skewed distribution are presented as median and interquartile ranges. Categorical data are presented as *n* (%). Baseline characteristics of the study participants were reported according to sgp130 serum quartiles: quartile boundaries (ng/ml) Q1: ≤452; Q2: >452 to ≤566; Q3: >566 to ≤705; Q4: >705.5.

Sgp130 serum levels (ng/ml) were not normally distributed therefore they were log transformed for the genetic association analysis. All genetic variants present in the combined CardioMetabo-Immuno chip were tested for association with log transformed serum sgp130 levels using a linear regression analysis under the assumption of an additive model of inheritance. A *p* value ≤ 1 × 10^−5^ was chosen as the a priori significance threshold. A suggestive association threshold was defined as *p* value > 1 × 10^−5^ ≤ 1 × 10^−4^. Two SNP pairs showed a high pairwise LD (*r*^2^ ≥ 0.8), rs9898140/rs4795780, and rs12884892/rs12886000, therefore only one SNP in the pair is reported in the analysis. Results are reported as beta (*β*) and standard error (SE) after adjustment for age, gender, and population structure (using MDS1, MDS2, and MDS3). MDS1 is highly correlated with latitude (*r* = 0.92, *p* < 0.0001). The variance in sgp130 levels explained by each SNP was estimated by partial *r*^2^, while the total variance explained by all the identified SNPs was estimated by *r*^2^.

The potential effect of SNP genotype on tissue expression (eQTL) of genes is reported from data published on the GTEx (https://gtexportal.org/home/) [52].

In a secondary analysis, we attempted to investigate if SNPs potentially relevant in the regulation of circulating sgp130 levels were associated with log transformed c-IMT baseline measures using the general linear model. We used two different models: model 1 adjusted for age, sex, and MDS1-3 and model 2 as per model 1, with addition of sgp130 as covariate. Results are reported as *β* and SE.

Standard epidemiological analyses were performed using SAS version 9.4 (SAS Institute, Cary, NC). Genetic association analysis was performed using Plink v1.07 [50].

## Supplementary information

Supplementary data file
