# Supplementary material for: Analysis of the genetic variants associated with circulating levels of sgp130. Results from the IMPROVE study
Source: Genes Immun. 2020 Jan 14;21(2):100–8. doi: 10.1038/s41435-019-0090-z (PMC7182533; doi:10.1038/s41435-019-0090-z)
Supplement: Supplementary file 1 — Supplementary data file [file 41435_2019_90_MOESM1_ESM.pdf]

Supplementary data file.

Analysis of the genetic variants associated with circulating levels of sgp130.

Results from the IMPROVE study.

Alice Bonomi [1], Fabrizio Veglia [1], Damiano Baldassarre [1,2], Rona J. Strawbridge [3,4], Zahra Golabkesh[5],Bengt Sennblad [6] Karin Leander [7], Andries J. Smit [8], Philippe Giral [9], Steve E. Humphries [10], Elena Tremoli [1], Anders Hamsten[4], Ulf de Faire [7], Bruna Gigante[,4] on behalf of the IMPROVE study group*.

[1] Centro Cardiologico Monzino, IRCCS, Milan, Italy; [2] Department of Medical Biotechnology and Translational Medicine, Università degli Studi di Milano; [3] Institute of Health and Wellbeing, University of Glasgow, Glasgow, UK; [4] Cardiovascular Medicine Unit, Department of Medicine Solna, Karolinska Institutet, Stockholm, Sweden; [5] Unit of Translational Epidemiology, Institute of Environmental Medicine, Karolinska Institutet, Stockholm, Sweden; [6] National Bioinformatics Infrastructure Sweden, Science for Life Laboratory, Uppsala University, Uppsala, Sweden;; [7] Unit of Cardiovascular and Nutritional Epidemiology, IMM; Karolinska Institutet, Stockholm, Sweden; [8] Department of Medicine, University Medical Center Groningen and University of Groningen, The Netherlands; [9]Assistance Publique-Hopitaux de Paris, Service Endocrinologie-Metabolisme, Groupe Hôpitalier Pitie-Salpetriere, Unités de Prévention Cardiovasculaire, Paris, France [10] Centre for Cardiovascular Genetics, University College London, United Kingdom.

Contents

1. Ethics
2. Supplementary Figures
    a. Supplementary Figure I
    b. Supplementary Figure II
3. Sample size consideration

1. **Ethics**

The IMPROVE study was funded by the Vth European Union (EU) programme, which involves seven recruiting centres in five European countries: Finland, France, Italy, the Netherlands, and Sweden. The study was designed in accordance with the rules of Good Clinical Practice (GCP), and with the ethical principles established in the Declaration of Helsinki. The study was approved by the IRB at each one of the seven recruiting centers: (1) the Regional Ethics Review Board at Karolinska Institutet, Stockholm Sweden, (2) IRB at the Groupe Hôpitalier Pitie-Salpetriere, Paris, France, (3) the IRB Comitato Etico delle Aziende Sanitarie della regione Umbria, Perugia and (4) the IRB at the Ospedale Niguarda Ca´Granda, Milano, both in Italy, (5) the IRB at the University Hospital Groningen, Groningen, the Netherlands, (6) the IRB Hospital District of Northern Savo and (7) and the IRB at University of Eastern Finland, both in Kuopio, Finland. Each participant provided two different written consents one for general participation in the study and one for genotyping.

2.1 Supplementary Figure I. Flowchart summarizing the IMPROVE study participants included in the present study.

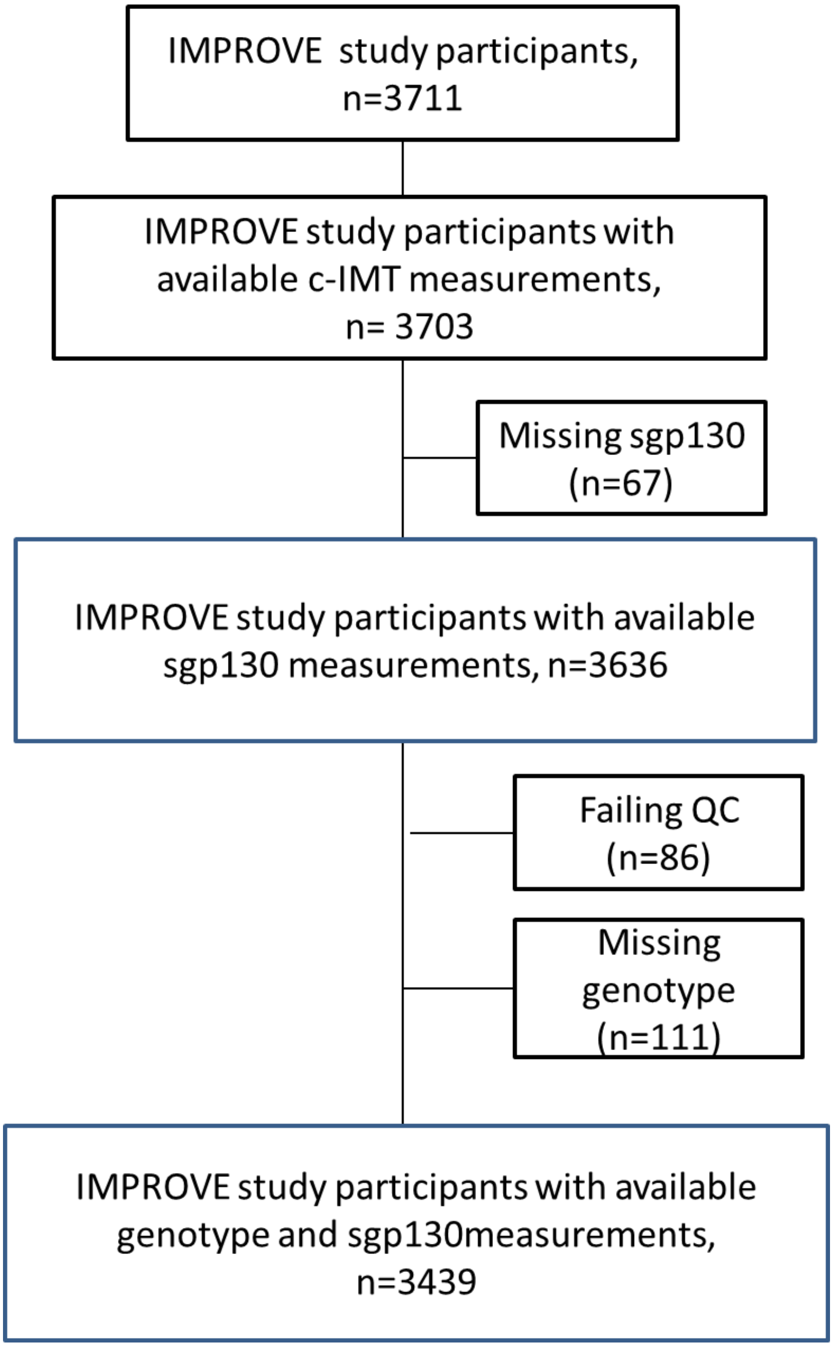

2.2 Supplementary Figure II. Manhattan plot sumaring the results of the association analysis

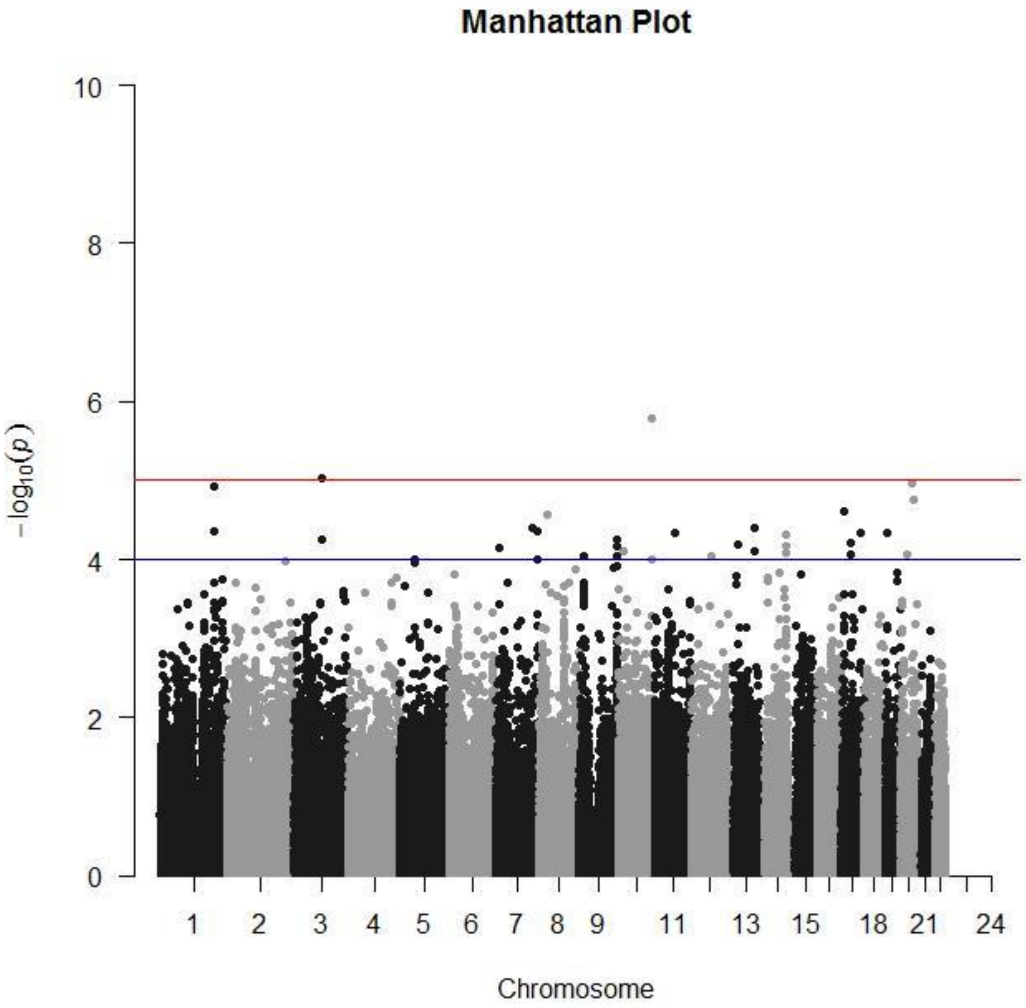

3. Sample size consideration: sample size 3400 subjects was sufficient to deem as significant with alpha=$1*10^{-5}$ and power=80% a partial correlation coefficient (correct for age, gender and population structure) of at least 0.09 between sgp130 levels and any SNPs analyzed.
